# Malicious Network Traffic Detection Based on Deep Neural Networks and Association Analysis

**DOI:** 10.3390/s20051452

**Published:** 2020-03-06

**Authors:** Minghui Gao, Li Ma, Heng Liu, Zhijun Zhang, Zhiyan Ning, Jian Xu

**Affiliations:** 1 China NARI Group Corporation (State Grid Electronic Power Research Institute), Nanjing 211106, China; gaominghui@sgepri.sgcc.com.cn (M.G.); mali@sgepri.sgcc.com.cn (L.M.); zzj519@163.com (Z.Z.); nzy@sgepri.sgcc.com.cn (Z.N.); 2 Beijing Kedong Electric Power Control System Co., Ltd., Beijing 100192, China; 3 Software College, Northeastern University, Shenyang 110169, China; l525309178@126.com

**Keywords:** network traffic, deep neural networks, Apriori association algorithm, anomaly detection

## Abstract

Anomaly detection systems can accurately identify malicious network traffic, providing network security. With the development of internet technology, network attacks are becoming more and more sourced and complicated, making it difficult for traditional anomaly detection systems to effectively analyze and identify abnormal traffic. At present, deep neural network (DNN) technology achieved great results in terms of anomaly detection, and it can achieve automatic detection. However, there still exists misclassified traffic in the prediction results of deep neural networks, resulting in redundant alarm information. This paper designs a two-level anomaly detection system based on deep neural network and association analysis. We made a comprehensive evaluation of experiments using DNNs and other neural networks based on publicly available datasets. Through the experiments, we chose DNN-4 as an important part of our system, which has high precision and accuracy in identifying malicious traffic. The Apriori algorithm can mine rules between various discretized features and normal labels, which can be used to filter the classified traffic and reduce the false positive rate. Finally, we designed an intrusion detection system based on DNN-4 and association rules. We conducted experiments on the public training set NSL-KDD, which is considered as a modified dataset for the KDDCup 1999. The results show that our detection system has great precision in malicious traffic detection, and it achieves the effect of reducing the number of false alarms.

## 1. Introduction

The internet processes a large number of users’ private data, which are vulnerable to various attacks from internal and external intruders [1]. With the development of internet technology, network attacks are becoming more frequent, and attack modes are becoming more diverse. Cyber attacks can lead to data breaches and cause huge economic losses. For example, Facebook lost hundreds of millions of dollars in data breaches due to hacking. In order to cope with increasingly complex network attacks, more and more security methods [2,3,4,5,6,7] analyze the characteristics of network traffic from different angles to establish a flexible and reliable intrusion detection system (IDS). The IDS collects and analyzes information about several key points in a computer network or computer system, and it analyzes whether the network system is being attacked. According to the difference in information sources, the IDS system can be divided into the network-based intrusion detection system (NIDS) and the host-based intrusion detection system (HIDS). The NIDS collects network behavior through network devices (switches, routers, and network taps), and it analyzes and identifies hidden attacks in network traffic. The HIDS uses various log files to detect attacks. Log files are collected by local sensors. Logs include sensor logs, system logs, software logs, file systems, disk resources, user account information, and so on. The IDS is of great significance for ensuring the security of network systems.

The NIDS is widely used, and it needs to collect and analyze a large number of network traffic data. These data hide the information about whether the detected traffic is benign or malicious. Learning algorithms can learn effective feature information from these data. According to different learning algorithms used, the NIDS systems can be divided into NIDS based on traditional machine learning algorithms and NIDS based on deep learning technology.

### 1.1. NIDS Based on Traditional Machine Learning

At present, most studies use standard public datasets KDD-Cup 99 and NSL-KDD for research; meanwhile, a variety of detection schemes were proposed using traditional machine learning algorithms. Wang et al. [8] proposed a decision tree-based intrusion detection algorithm. In their experiments, the C4.5 algorithm could achieve great detection accuracy, but there was still a certain error rate. Subsequently, Farid et al. [9] studied a technique of implementing feature selection from training datasets to achieve intrusion detection. They used ID3 and C4.5 decision tree algorithms to calculate the weight values of each feature. The basis for determining weight was the minimum depth of the feature node in the decision tree. When the feature did not appear in the decision tree, the allocation weight was zero. Reference [10] designed an intrusion detection algorithm using AdaBoost technology, which uses a decision tree as a basic classifier. Their detection scheme had a lower false alarm rate and a higher detection rate, but it could not use incremental learning methods. In Reference [11], intrusion detection was performed using different machine learning methods. After comparative experiments, it was found that the performance of the decision tree classifier was better than that of the support vector machine (SVM) learning algorithm. Reference [12] proposed a two-level classification detection algorithm, which used naïve Bayes as a basic classifier in the first stage and used nominal-to-binary filtering and cross-validation for testing in the second stage. Reference [13] further optimized the two-level detection algorithm. They used balanced nested dichotomy in the first stage and random forest for prediction classification in the second stage. Because the enhancement of the two-level algorithm resulted in a higher detection rate and a lower false alarm rate, subsequent academic research proposed a variety of two-level detection algorithms, including the use of principal component analysis (PCA) dimensionality reduction, SVM support vector machines, and other related technologies.

### 1.2. NIDS Based on Deep Learning Technology

Traditional machine learning algorithms can learn local features of network traffic, but they cannot learn deep features of network traffic. The IDS detection effect based on traditional learning algorithms has room for improvement. The deep neural network (DNN) is a complex sub-item of machine learning. It can learn deep feature information in data through multiple hidden layers, including some hierarchical features and hidden order relationships. In recent years, deep learning technology achieved significant results in the fields of computer vision [14], medical analysis [15], natural language processing [16], etc. Network security researchers are beginning to apply deep learning technology to intrusion detection systems. There exists an order relationship within network traffic, and recurrent neural networks (RNN) can record previous information and act on the current output. 

Therefore, Reference [17] proposed a network intrusion detection architecture based on novel feature selection model and recurrent neural networks (RNNs). Their architecture included four steps. The final step was to evaluate various IDS RNN models based on two things. The first task was a comparison with other IDS classifiers. The second involved measurement memory profilers of the proposed models. Finally, the results showed that the IDS using RNN had better performance. Considering the significant achievements of the convolutional neural network (CNN) in the field of computer vision, Reference [18] studied the performance of CNN technology in intrusion detection. They modeled network traffic as a time series and used supervised learning methods to model and analyze Transmission Control Protocol/Internet Protocol (TCP/IP) packets over a predefined time frame. The results showed that the performance of the CNN and its variant architectures were superior to traditional machine learning algorithms. Reference [19] evaluated the effectiveness of various shallow and deep networks in intrusion detection applications. They trained and evaluated shallow and deep networks on the KDD-Cup 99 and NSL-KDD datasets in two-class and multi-class situations, and they obtained that deep networks have better performance than shallow networks in most experimental environments. Reference [20] proposed a deep learning scheme, called dilated convolutional autoencoders (DCAEs), for the network intrusion detection model, which combined the advantages of stacked autoencoders and CNNs. This model could automatically learn essential features from large-scale and more varied unlabeled raw network traffic data consisting of real-world traffic from botnets, web-based malware, exploits, APTs (advanced persistent threats), scans, and normal traffic streams.

Our main contributions can be summarized as follows: we studied how to preprocess network traffic data in preparation for deep neural networks and association analysis algorithms, in order to conduct a better analysis. We compared the effects of various neural networks in intrusion detection, based on public datasets. Finally, we selected DNN-4 with great performance as the main part of this system. Association analysis was performed on the classified data to filter out normal traffic that is misclassified as malicious traffic. We found that, after filtering with the association rule, the precision of intrusion detection showed great improvement. Finally, we designed a novel anomaly detection system based on a deep neural network and association analysis.

## 2. Proposed Malicious Network Traffic Detection System

The traditional anomaly detection system performs feature matching detection on network traffic based on an existing expert knowledge base, which has some drawbacks such as low accuracy and many false positives. Our system is based on a deep neural network and association analysis. The deep neural network can mine the deep feature information of network traffic, as well as classify unknown attack traffic. Because the deep neural network still has a slight false rate, after classification, we filter the classified traffic through feature matching to remove the normal traffic that is misclassified as malicious traffic. During the process of feature matching, we use the association rules corresponding to the “normal” label, which are calculated by the Apriori association algorithm. 

**Figure 1:**
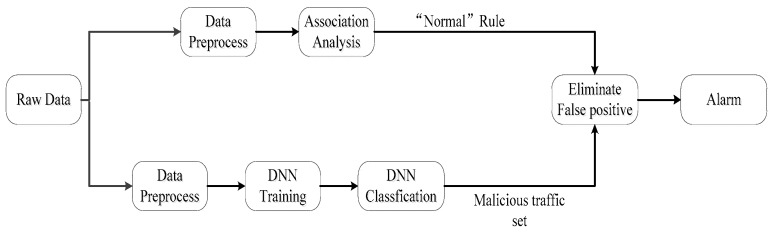
The process of our anomaly detection system, which mainly consists of preprocessing the dataset, deep neural network (DNN) training, classification, and association analysis.

The proposed system in this paper mainly consists of three steps. Step one is the preprocessing of the dataset. Step two is DNN training and classification. Step three is association analysis. The process of our system is shown in Figure 1. Firstly, we perform preprocessing operations on the raw traffic data, because traffic data are described by many characteristics, but the input of the DNN model and Apriori association analysis require different data formats. Secondly, we train the DNN model and use the trained model to classify network traffic. At the same time, the Apriori association algorithm is used to mine the association rules between the discretized features and the ‘normal’ label hidden in the dataset. Subsequently, the mining association rules are applied to match the classified malicious traffic set and filter out the misclassified normal traffic. Finally, alarm logs are generated, which are described by some characteristics. Below, we introduce the main technical parts of data preprocessing, model training, classification, and association analysis in detail.

### 2.1. Data Preprocessing

Because the DNN model can only process digital information and the Apriori algorithm is suitable for mining association rules between discretized features, the corresponding preprocessing operations are firstly performed before the data are input into the neural network and the association analysis is performed. 

#### 2.1.1. Data Preprocessing Corresponding to DNN Models

The data preprocessing methods affect the training of the model and the classification effect of the model. There are different preprocessing methods for different datasets, because each data preprocessing method has its own limitations. Intrusion detection is a classification task. Before entering the DNN model, we mainly use numerical methods, one-hot coding, and three standardized processing methods for the network traffic dataset. We introduce them separately below.

Numerical method: The network traffic data usually contain some character-type characteristics, such as the protocol type (TCP, UDP, ICMP) of the network traffic, the network service type of the target host, and the state of the network connection (normal or error). These character-type attribute values cannot be directly input into the DNN model; thus, they need to be converted into numeric forms. For example, the network protocol type includes three discretized attribute values: TCP, UDP, and ICMP. We digitize it into 1, 2, and 3 to conform to the input format of the DNN model.

One-hot encoding: The optimization algorithm used in DNN model training is calculated based on the metric in the vector space. One-hot encoding allows the non-partially ordered relationship variables to maintain their characteristics and reach the point. The distance is equidistant. For example, in the numerical process, TCP, UDP, and ICMP are converted to 1, 2, and 3. Although they conform to the input format of the model, there is still a certain size or order relationship. In reality, the three feature values have no ordering relationship or size relationship. Therefore, it needs to be subjected to one-hot encoding processing as 001, 010, and 100. One-hot encoding expands the characteristics of the data to a certain extent, i.e., the value of each feature after processing is only 0 or 1, which is more conducive to the calculation of the distance between features or calculation of similarity.

Standardization: Standardization can eliminate the differences between various features and accelerate the convergence of neural network weight parameters. For example, the network traffic data have the following two characteristics: (1) the byte number of the data from the source host to the target host is between 0 and 1,379,963,888, and (2) the number of failed login attempts is set to [0, 5]. These two features have different dimensions and orders of magnitude. If used directly, one of the features will have a greater impact on the effect of the network model and the other feature will have a smaller impact. Through the standardization process, different feature variables can conform to a standard normal distribution, i.e., the mean value is 0 and the standard deviation is 1, such that the prediction effect of the neural network model will be controlled by multiple feature variables of the same size. The standardized formula is shown in Equation (1), where μ is the average of all sample data and σ is the standard deviation of all sample data.

(1) $$x^{*}=\frac{x-\mu }{\sigma }$$

#### 2.1.2. Data Preprocessing Corresponding to Apriori Association Algorithm

Apriori is mainly used to calculate the association rules between discretized features. Therefore, we delete a large number of continuous features included in the network traffic features. Only the discretized features and label features are saved for subsequent association rule mining. The processed results are shown in Table 1.

### 2.2. DNN Training and Classification

DNN is a deep learning model, which consists of an input layer, many hidden layers, and an output layer. The DNN model can learn the abstract high-dimensional feature representation of the network traffic data by passing the network traffic data into the hidden layer, which can adapt to the changing network environment, and the detection effect is better than the traditional IDS system based on rule matching. Current research [21] shows that a deep neural network composed of fully connected layers is sufficient to perform well on various publicly available datasets, and it can achieve great recognition accuracy with a short training time. DNN can be expressed as a mapping relationship *O: R^m^ × R^n^*, where *m* represents the size of an input item, x = x_1_, x_2_, …, x_m-1_, x_m_, and *n* indicates the size of an output item *O*(*x*). We use a four-layer deep neural network DNN consisting of fully connected layers that perform well in detection. Based on the preprocessing method proposed in this paper, our input layer uses 122 neurons instead of 41. 

We chose the best network structure from DNN-3, DNN-4, DNN-5, and DNN-6 for training an IDS model with NSL-KDD. Finally, the structure of our DNN model was as shown in Table 2.

We can adjust the number of neurons in the output layer according to the network traffic, i.e., into two categories or five categories. When the model only classifies network traffic into normal traffic and malicious traffic, the final output layer uses one neuron. When the model needs to classify the network traffic into five types (Normal, Prob, Dos, U2R, and R2L), the output layer uses five neurons. The loss function (cross-entropy loss function) used in the two classifications is shown in Equation (2). When the classification is multi-class, the loss function in Equation (3) is used, where *n* is the number of samples, and *m* is the number of categories, which is a multi-output loss function.

(2) $$loss=-\sum_{i=1}^{n}{\hat{y}}_{i}\log y_{i}+(1-{\hat{y}}_{i})\log(1-{\hat{y}}_{i})$$

(3) $$loss=-\sum_{i=1}^{n}{\hat{y}}_{i1}\log y_{i1}+{\hat{y}}_{i2}\log y_{i2}+L+{\hat{y}}_{im}\log y_{im}$$

**Figure 2:**
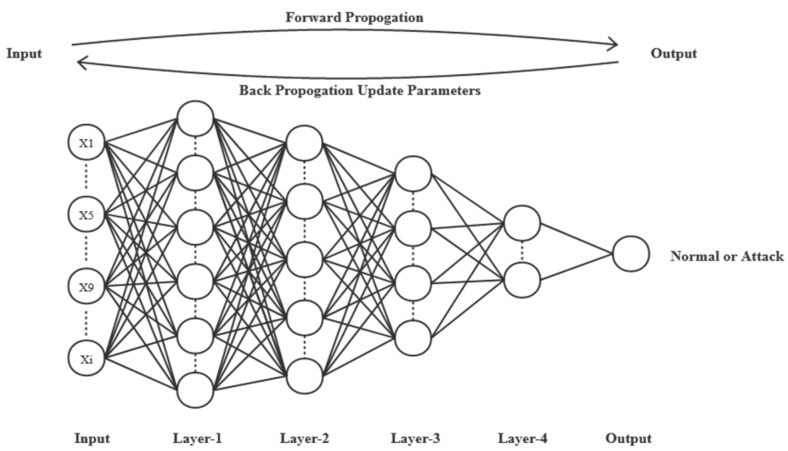
Training process of DNN: binary classification.

During the training process, we use the stochastic gradient descent algorithm (SGD) to iteratively update the weight parameters of each layer so as to continuously improve the accuracy of the model prediction. The model training process of binary classification is shown in Figure 2.

### 2.3. Association Analysis

As one of the earliest methods used in intrusion detection, the association analysis algorithm is of great significance. Previous research applied association analysis methods to the automatic expansion of intrusion detection rule bases, i.e., the current association analysis methods are mostly used in misuse detection methods. The association analysis method can be used to calculate the rules that meet the requirements according to the set support and confidence, as well as shorten the discovery cycle of the rules. The Apriori algorithm can mine the inherent relationship between variables. Based on this idea, we use the Apriori algorithm to mine the inherent relationship between the discretized characteristics of network traffic and the “normal” label (association rules), then use this rule to perform feature matching on the network traffic classified by the DNN model, in order to filter out the normal traffic that is misclassified.

The Apriori algorithm is a classic association analysis algorithm. Its main function is to mine the relationships between different variables and summarize useful rule information. The steps for mining association rules using the Apriori algorithm are as follows:

**Step 1:** Calculate frequent item sets

When the number of variables (items) in the calculated set is greater than 0:

Build a list of candidate sets of *k* variables.
Examine the data to determine whether each item set is a frequent item set (the support of the item set is greater than the set minimum support of 0.5).
If the item set is frequent, keep the item set and build a list of candidate sets consisting of *k* + 1 items.

This is shown in Equation (4).

(4) {Variable A,Variable B,Variable C…}

**Step 2:** Construct association rules, i.e., based on all the frequent item sets that were mined, construct rules with a confidence level greater than 80% and related to the “normal” label, as shown in Equation (5).

(5) $$\left\{Frequent\;item-set\;E\}\Rightarrow \{Frequent\;item-set\;F(include^{\prime \prime }Normal^{\prime \prime }variable)\right\}$$

After mining the association rules related to the “normal” label based on the Apriori algorithm, we use the association rule consisting of the frequent item set E, which has the most variables with the highest credibility, as the feature matching rule in the process of eliminating false positives, which can guarantee the maximum efficiency of filtering out normal traffic that is misclassified as malicious traffic. 

After the false positives are eliminated, we generate alarm logs for network traffic which is predicted as malicious.

## 3. Experimental Results and Analysis

The proposed system was mainly tested on the existing public dataset NSL-KDD. Firstly, we compared the single DNN model with other learning models in the datasets NSL-KDD and CICIDS 2017. It can be known from experiments that the DNN model was one of the learning models that performed well. Finally, we tested the system of combining neural networks with association analysis on the dataset NSL-KDD, and we found that it is more precise than malicious traffic detection using a single DNN. This chapter introduces the experimental steps in detail.

### 3.1. NSL-KDD Dataset Preprocessing

The NSL-KDD [22] dataset is widely used in intrusion detection game data provided by Lincoln Labs. The training set contains 125,973 network traffic records, and the test set contains 22,544 network traffic records. Each connection in the dataset is described with 41 characteristics, as shown in Table 3.

The features of each network traffic record include a total of 42 features, including three character discretized features, six numeric discretized features, 32 numeric continuous features, and one category label. In addition to normal or attack, the type of anomaly can also be further divided into four categories: Probe (scan and detect), Dos (denial of service attack), U2R (illegal access), and R2L (unauthorized remote access); thus, this dataset can be tested in two or five categories. The test set contained some attack types that did not appear in the training set so that the generalization ability of the intrusion detection system could be tested. The information on each network traffic in the dataset was saved in CSV format. The following is a description of the initial traffic in the dataset:

{2, tcp, smtp, SF, 1684, 363, 0, 0, 0, 0, 0, 1, 0, 0, 0, 0, 0, 0, 0, 0, 0, 0, 1, 1, 0.00, 0.00, 0.00, 0.00, 1.00, 0.00, 0.00, 104, 66, 0.63, 0.03, 0.01, 0.00, 0.00, 0.00, 0.00, 0.00, normal},

Where the first 41 items of each traffic includes characteristic information, and the last item includes category label information.

#### 3.1.1. Data Preprocessing Corresponding to the DNN Model

We used the pandas model to read the data information from the CSV file, and then numerically processed, one-hot encoded, and standardized the data. The final processed data are shown below, where each traffic used 122 features as input to the DNN model.

{−0.1555340871−0.0219881116 −0.0968959693 −0.0176238573 −0.0591039397 −0.0194592491 −0.1135211665 −0.1439989462 −0.8903726296 −0.016493558 −0.0494534133 −0.0126377698 ...}

#### 3.1.2. Data Preprocessing Corresponding to Apriori Algorithm

After reading the traffic information from the CSV file using the pandas model, the continuous features in the data were deleted, and only the discretized features and label information of the network traffic were kept, as shown in Table 4.

### 3.2. Training and Prediction of DNN

#### 3.2.1. Evaluation Metrics

The metrics such as accuracy, precision, recall, and f-score are often used to measure the performance of data mining algorithms on a minority class. In addition, the true positive (TP) can be expressed as an attack occurring with an alarm raised, the false positive (FP) can be expressed as no attack occurring with an alarm raised, the true negative (TN) can be expressed as no attack occurring with no alarm raised, and the false negative (FN) can be expressed as an attack occurring with no alarm raised. The accuracy, precision, recall, and f-score can be defined as follows:

(6) $$\mathrm{Accuracy}=\frac{\mathrm{TP}+\mathrm{TN}}{\mathrm{TP}+\mathrm{TN}+\mathrm{FP}+\mathrm{FN}}$$

(7) $$\mathrm{Precision}=\frac{\mathrm{TP}}{\mathrm{TP}+\mathrm{FP}}$$

(8) $$\mathrm{Recall}=\frac{\mathrm{TP}}{\mathrm{TP}+\mathrm{FN}}$$

(9) $$F1-\mathrm{Score}=\frac{\mathrm{Precision}\times \mathrm{Recall}}{\mathrm{Precision}+\mathrm{Recall}}$$

#### 3.2.2. Comparison of Different Neural Networks

We used the DNN model composed of a fully connected layer and dropout layer as a neural network model for intrusion detection. During training, the loss functions “binary_crossentropy” and “category_crossentropy” were used for the second and fifth classifications, respectively, in “Keras”. The “SGD” optimizer was used as the ultimate optimizer, and the batch size was set to 32. Considering that the number of different hidden layers affects the classification effect of the DNN, we trained DNN models with three, four, five, and six hidden layers and performed binary-class and five-class detection comparison analysis on the test set, as shown in Table 5 and Table 6.

In our system, the most important measures were accuracy and precision, having a low false positive rate. Therefore, we firstly paid attention to precision, which can be expressed as the ratio of samples with correct predictions to the total number of samples predicted. From Table 5, we can find that DNN-4 achieved the highest accuracy of 82.74%, despite its precision being 0.88, which was lower than others; this can be raised via the eliminating process. From Table 6, the results show that the DNN-4 model could also reach the highest accuracy rate of 77.03%. As for different attack types, such as Dos, Probe, R2L, and U2R, the precision of DNN-4 was similar to that of DNN-5, while DNN-6 also had a great effect. However, as the number of layers increased, the prediction accuracy rate did not improve. For example, for the DNN models with five layers and six layers, the prediction accuracy was 76.22% and 75.99%. Because each layer of DNN consists of fully connected layers, if there are too many layers, there will be a large number of weight parameters that need to be adjusted, increasing the difficulty of training. On the other hand, models such as SVM and random forest had lower accuracy. Based on our data preprocessing method, DNN-4 was better than the other models. Therefore, we finally adopted a DNN structure with four hidden layers for intrusion detection classification.

We also carried out comparison experiments with other learning models using dataset CICIDS 2017 [23]. It can be found that DNN-4 was also among the best-performing models, as shown in Table 7. The accuracy of DNN-4 was above 95%. 

### 3.3. Mining Association Rules

After the network traffic data were pre-processed correspondingly, we used the Apriori algorithm to calculate the association rule corresponding to “normal” to prepare for the next step of eliminating false positives. Each network traffic record in the dataset NSL-KDD had a total of nine discretized features, including “protocol_type”, “service”, “flag”, “land”, “logged_in”, “root_shell”, “su_attempted”, “is_hot_login”, and “is_guest_login”. The Apriori algorithm is suitable for mining association rules between discretized variables (features). We firstly calculated the frequent item sets with the support of more than 0.5 in various combinations of these nine discretized features and label features. Some of the calculated frequent item sets can be seen in Table 8.

After calculating all the frequent item sets that met the minimum support threshold, we constructed rules with a greater than 80% confidence level related to the “normal” label based on the existing frequent item sets; these rules are shown in Table 9.

It can be seen from the above table that there were multiple association rules related to the “normal” tag with credibility of more than 80%. For example, the first association rule can be interpreted as if the network traffic has the characteristic “SF”, which can be inferred as normal traffic; the reliability for this rule was 84.5%. Because we needed to use the mining association rules to perform feature matching, so as to filter classified network traffic and reduce the rate of false positives, for the most effective filtering, we only used the fourth rule for feature matching, because its predecessor set contained multiple features to achieve stricter feature matching for filtering.

### 3.4. Evaluation Results of Elimination

The goal of our system was to have high precision and low false alarms. Firstly, we used the four-layer DNN model to predict the test set to categorize the normal traffic set and the malicious traffic set, and then we used “normal” association rules to perform feature matching on the malicious traffic set to filter out the misclassified normal traffic, before finally determining the malicious network traffic information and output of the alarm logs. 

#### 3.4.1. Binary Classification

Considering that intrusion detection mainly features a binary classification (i.e., attack or normal), our experiments showed that the “normal” rule had a better result when using a filter based on binary classification. The comparison of before and after filtering is shown in Table 10. According to Table 10, before the filtering was performed, the DNN-4 model predicted that the number of malicious traffic items was 11,753. After filtering using the “normal” association rule, the number of malicious traffic items was 7500, and 4253 traffic items were successfully filtered out. The corresponding precision rate for malicious traffic increased from 0.88 to 0.99. We found that the association rules could also be applied to DNN-3, DNN-5, and DNN-6, indicating that our system successfully filtered out most of the misclassified normal traffic using the association rules, meaning that it would generate fewer false alarms.

#### 3.4.2. Multi-class Classification

In the five-category classification experiments, we found that DNN-4 had higher accuracy on the NSL-KDD test-set, as can be seen from the result in Section 3.2. The comparison of before and after filtering is shown in Table 11. From this table, we can find that the number of malicious traffic items before filtering was 9863, and the number of malicious traffic items after filtering was 7336. Most of the filtered traffic was misclassified. The precision for Dos and Probe increased from 0.88 to 0.91 and from 0.72 to 0.89, respectively. Meanwhile, the precision of U2R was unchanged. This means that our system could still reduce false alarm rates in the case of multiple classifications.

From the results of binary classification and multi-class classification, it can be seen that the application of association analysis after classification had a great effect, especially for binary classification. This also means that the combination of association analysis and other classifiers can help reduce the number of false alarms generated by the IDS system. After classifying network traffic and eliminating false positives (filtering), we describe the malicious traffic information and generate corresponding alarm logs in Table 12.

## 4. Conclusions

We used deep neural network and association analysis technology to design an anomaly detection system. The system can detect, classify, and generate alarms for abnormal traffic in the network, with a lower false alarm rate. Based on the dataset NSL-KDD, we tested the binary classification of network traffic. The results showed that filtering using association rules could effectively improve the detection precision of the system. Moreover, our detection system also had a good detection effect in a multi-class experimental environment. This two-level detection system that classifies and then filters network traffic has higher precision and fewer false positives in terms of detection results. This indicates that the application of association analysis and a deep neural network has a good effect. The structure of the deep neural network still has a lot of room for optimization, and increasing the precision rate while maintaining recall rate remains a challenge, which can be addressed in future work.

## Figures and Tables

**Table 1:** The results of data preprocessing of the Apriori association algorithm.

Land	Logged_in	Protocol_type	Service	…	Label
land	logged_in	UDP	aol	…	Attack
no_land	no_logged_in	TCP	auth	…	Normal

**Table 2:** The architecture of DNN-4.

Layers	Type	Output Shape	Number of Units	Activation Function
Input		(None, 122)	122	
0–1	Fully connected	(None, 256)	256	ReLU
1–2	Dropout (0.01)			
2–3	Fully connected	(None, 256)	256	ReLU
3–4	Dropout (0.01)			
4–5	Fully connected	(None, 256)	256	ReLU
5–6	Dropout (0.01)			
6–7	Fully connected	(None, 256)	256	ReLU
7–8	Dropout (0.01)			
Output		(None, 1 or 5)		

**Table 3:** The characteristics of NSL-KDD, which include four parts.

No	Characteristic
1	Basic characteristics of TCP connection (9 types, 1–9)
2	TCP connection content characteristics (13 types, 10–22)
3	Statistical characteristics of time-based network traffic (9 types in total, 23–31)
4	Statistical characteristics of host-based network traffic (10 in total, 32–41)

**Table 4:** The results of data preprocessing corresponding to the Apriori algorithm.

Land	Logged_in	Root_shell	Su_attempted	Is_host_login	Is_guest_login	Protocol_type	Service	Flag	Label
no_land	no_logged_in	no_root_shell	no_su_attempted	no_is_host_login	no_is_guest_login	tcp	ftp_data	SF	normal
no_land	no_logged_in	no_root_shell	no_su_attempted	no_is_host_login	no_is_guest_login	udp	other	SF	normal
no_land	no_logged_in	no_root_shell	no_su_attempted	no_is_host_login	no_is_guest_login	tcp	private	S0	attack
no_land	logged_in	no_root_shell	no_su_attempted	no_is_host_login	no_is_guest_login	tcp	http	SF	normal
no_land	logged_in	no_root_shell	no_su_attempted	no_is_host_login	no_is_guest_login	tcp	http	SF	normal
no_land	no_logged_in	no_root_shell	no_su_attempted	no_is_host_login	no_is_guest_login	tcp	private	REJ	attack
no_land	no_logged_in	no_root_shell	no_su_attempted	no_is_host_login	no_is_guest_login	tcp	private	S0	attack
no_land	no_logged_in	no_root_shell	no_su_attempted	no_is_host_login	no_is_guest_login	tcp	private	S0	attack
no_land	no_logged_in	no_root_shell	no_su_attempted	no_is_host_login	no_is_guest_login	tcp	remote_job	S0	attack

**Table 5:** Effects of different DNNs on binary classification, based on NSL-KDD test set. RNN—recurrent neural network; CNN—convolution neural network; RF—random forest; SVM—support vector machine.

Model	Accuracy	Precision	Recall	F-score
DNN-3	79.76%	0.91	0.72	0.80
DNN-4	82.74%	0.88	0.81	0.84
DNN-5	81.33%	0.92	0.74	0.82
DNN-6	80.56%	0.90	0.74	0.81
RNN	77%	0.95	0.63	0.76
CNN4	80%	0.96	0.67	0.79
RF	77%	0.95	0.63	0.76
SVM	78%	0.91	0.68	0.77

**Table 6:** Effects of different models on multi-class classification, based on NSL-KDD test set.

Model		DNN-3	DNN-4	DNN-5	DNN-6	RNN	CNN4	RF	SVM
	Accuracy	76.84%	77.03%	76.22%	75.99%	71.34%	73.58%	46.73%	72.13%
Normal	Precision	0.71	0.72	0.70	0.70	0.66	0.66	0.63	0.67
	Recall	0.94	0.94	0.94	0.94	0.84	0.95	0.97	0.86
	F1-score	0.81	0.81	0.80	0.80	0.74	0.78	0.77	0.75
Dos	Precision	0.91	0.88	0.91	0.91	0.80	0.96	0.00	0.81
	Recall	0.85	0.86	0.83	0.82	0.84	0.76	0.00	0.84
	F1-score	0.88	0.87	0.87	0.86	0.82	0.85	0.00	0.83
Probe	Precision	0.71	0.72	0.70	0.69	0.71	0.61	0.25	0.72
	Recall	0.75	0.75	0.74	0.76	0.67	0.65	0.48	0.65
	F1-score	0.73	0.73	0.72	0.72	0.69	0.63	0.33	0.69
R2L	Precision	0.00	0.27	0.31	0.42	0.19	0.95	0.00	0.07
	Recall	0.00	0.00	0.00	0.00	0.00	0.06	0.00	0.00
	F1-score	0.00	0.00	0.01	0.01	0.00	0.11	0.00	0.00
U2R	Precision	0.50	0.62	0.67	0.80	0.28	0.00	0.00	0.21
	Recall	0.10	0.07	0.09	0.12	0.07	0.00	0.00	0.15
	F1-score	0.17	0.13	0.16	0.21	0.12	0.00	0.00	0.17

**Table 7:** Comparison of the effects of different models based on CICIDS 2017 dataset.

Model		DNN-3	DNN-4	DNN-5	DNN-6	RNN	CNN4	RF
SSH	Accuracy	99.17%	99.03%	98.61%	97.72%	97.36%	96.89%	99.98%
	Precision	0.93	0.81	0.72	0.50	0.00	0.00	1.00
	Recall	0.50	0.85	0.98	0.24	0.00	0.00	1.00
	F1-score	0.65	0.83	0.83	0.32	0.00	0.00	1.00
FTP	Accuracy	99.17%	99.03%	98.61%	97.72%	97.36%	96.89%	99.98%
	Precision	0.94	0.80	0.73	0.94	0.88	0.00	1.00
	Recall	1.00	0.98	0.79	0.50	0.31	0.00	1.00
	F1-score	0.97	0.88	0.76	0.65	0.46	0.00	1.00
Web	Accuracy	98.22%	98.68%	97.34%	89.01%	98.64%	98.69%	99.96%
	Precision	0.16	0.00	0.32	0.09	0.49	0.00	1.00
	Recall	0.08	0.00	0.91	0.84	0.83	0.00	0.97
	F1-score	0.01	0.00	0.47	0.17	0.62	0.00	0.99
Bot	Accuracy	96.94%	99.33%	99.01%	99.01%	99.01%	99.01%	99.91%
	Precision	0.11	0.68	0.00	0.00	0.00	1.00	0.99
	Recall	0.28	0.61	0.00	0.00	0.00	0.01	0.94
	F1-score	0.15	0.65	0.00	0.00	0.00	0.02	0.97
DDOS	Accuracy	98.44%	98.72%	98.08%	98.56%	95.18%	75.23%	99.98%
	Precision	0.98	0.98	0.97	0.98	0.93	0.70	1.00
	Recall	1.00	1.00	1.00	1.00	0.99	1.00	1.00
	F1-score	0.99	0.99	0.98	0.98	0.96	0.82	1.00
PortScan	Accuracy	97.43%	95.25%	98.77%	98.43%	87.64%	64.29%	99.99%
	Precision	0.96	0.92	0.96	0.97	0.82	0.61	1.00
	Recall	1.00	1.00	1.00	1.00	1.00	1.00	1.00
	F1-score	0.98	0.96	0.98	0.99	0.90	0.76	1.00

**Table 8:** Some of the calculated frequent item sets.

No	Item-set
1	{“normal”, “no_land”}
2	{“no_root_shell”, “normal”}
3	{“normal”, “no_su_attempted”}
4	{“normal”, “SF”}
5	{“no_is_host_login”, “normal”}
6	{“no_is_guest_login”, “normal”}

**Table 9:** Calculated association rules.

No	Association Rules
1	{“SF”} => {“normal”} conf: 0.845
2	{“no_land”, “no_su_attempted”, “SF”} => {“normal”} conf: 0.845
3	{“no_land”, “no_root_shell”, “no_su_attempted”, “SF”} => {“normal”} conf: 0.845
4	{“SF”, “no_is_host_login”, “no_su_attempted”, “no_root_shell”, “no_land”} => {“normal”} conf: 0.845

**Table 10:** Result of two classifications before and after filtering, based on the NSL-KDD test set.

Method	Precision	Recall	F-score	Number of Alarm Raised
(Before filtering) DNN-3	0.91	0.72	0.80	10,141
(After filtering) DNN-3	0.99	0.55	0.71	7165
(Before filtering) DNN-4	0.88	0.70	0.80	11,753
(After filtering) DNN-4	0.99	0.54	0.70	7500
(Before filtering) DNN-5	0.92	0.74	0.82	10,342
(After filtering) DNN-5	0.99	0.56	0.72	7300
(Before filtering) DNN-6	0.90	0.74	0.81	10,523
(After filtering) DNN-6	0.99	0.56	0.72	7301

**Table 11:** Result of five classifications before and after filtering, based on the NSL-KDD test set.

	Label	Precision	Recall	F-score	Number of Alarm Raised
Before filtering	Normal	0.72	0.94	0.81	9863
	Dos	0.88	0.86	0.87	
	Probe	0.72	0.75	0.73	
	R2L	0.27	0.00	0.00	
	U2R	0.62	0.07	0.13	
After filtering	Normal	0.63	0.99	0.77	7336
	Dos	0.91	0.73	0.81	
	Probe	0.89	0.49	0.63	
	R2L	0.30	0.00	0.00	
	U2R	0.62	0.07	0.13	

**Table 12:** Some of the generated alarms.

Protocol_type	Service	Flag	Urgent	Hot	Count	Prediction
tcp	private	REJ	0	0	255	Dos
tcp	private	REJ	0	0	255	Dos
tcp	private	REJ	0	0	255	Dos
tcp	telnet	S0	0	0	235	Dos
tcp	private	REJ	0	0	255	Dos
tcp	ldap	REJ	0	0	255	R2L
tcp	pop_3	S0	0	0	255	Dos
tcp	courier	REJ	0	0	255	Dos
tcp	discard	RSTO	0	0	255	Dos
tcp	http	RSTR	0	0	241	Dos
tcp	private	REJ	0	0	255	Probe
tcp	private	S0	0	0	255	Dos
tcp	mtp	REJ	0	0	255	Dos
tcp	telnet	S0	0	0	91	Dos
tcp	iso_tsap	REJ	0	0	255	Dos
tcp	private	REJ	0	0	255	Dos
tcp	other	REJ	0	0	255	Probe
tcp	telnet	REJ	0	0	106	Probe
tcp	private	REJ	0	0	255	Dos
tcp	smtp	S0	0	0	255	Dos
